# Differences in Trait Impulsivity Indicate Diversification of Dog Breeds into Working and Show Lines

**DOI:** 10.1038/srep22162

**Published:** 2016-03-10

**Authors:** Fernanda Ruiz Fadel, Patricia Driscoll, Malgorzata Pilot, Hannah Wright, Helen Zulch, Daniel Mills

**Affiliations:** 1 University of Lincoln, School of Life Sciences, Joseph Banks Laboratories, Lincoln, LN6 7DL, UK

## Abstract

Impulsiveness describes the inability to inhibit behaviour in the presence of salient cues. Trait-level impulsivity exists on a continuum and individual differences can be adaptive in different contexts. While breed related differences in behavioural tendency in the domestic dog (*Canis familiaris*) are well established, the phenomenon within lines of a breed which have been selected more recently is not well studied, although it may challenge the popular notion of breed-typical behaviour. We describe differences in impulsivity between and within two dog breeds with working and show lines selected for different levels of impulsivity: Border Collies (herding work) and Labrador Retrievers (gun work). Recent show line selection might have lessened differences in impulsivity between breeds. We tested this hypothesis on a dataset of 1161 individuals assessed using a validated psychometric tool (Dog Impulsivity Assessment Scale - DIAS). Collies were more impulsive on average, consistent with the original purpose of breed selection. Regarding line, working Collies differed from working Labradors, but show lines from the two breeds were not significantly different. Altered or relaxed artificial selection for behavioural traits when appearance rather than behaviour become the primary focus for breeders may reduce average differences in impulsivity between breeds in show lines.

Impulsivity is a behavioural trait described as the inability to delay reward gratification. There is evidence that this trait is stable in humans, dogs and other animals^1^,^2^,^3^,^4^. In dogs, impulsivity has been considered largely in relation to aggressive behaviour^5^, with higher levels of impulsivity associated with low levels and poor regulation of serotonin and dopamine^6^. In humans, it is related to violent behaviour and behavioural disorders, such as attention deficit hyperactivity disorder (ADHD)^1^,^7^. Research on behavioural traits has been used as an aid to animal management and welfare, as well as an aid in the selection of dogs for their various working roles^8^,^9^. Impulsivity can be related to behavioural problems which are the main reason for dogs to be rejected in selection for working functions and relinquished by pet owners^10^,^11^,^12^.

The study of behavioural traits in animals is of growing interest to a number of fields, for example as a tool to understand human personality traits in an evolutionary context as well as the lifetime costs and benefits of specific traits^4^. Dogs can be used as models to understand human personality and behavioural disorders, since their behaviour repertoire is simpler yet similar enough for comparable conclusions. A good example is canine Obsessive Compulsive Disorder (OCD), which is well defined by clinicians. Its complex genetics is starting to be elucidated in dogs and can be used in a comparative manner to guide human OCD research^13^. Many aspects of behaviour seem to be analogous between humans and dogs, possibly as a result of convergent evolution through domestication and shared social experience during development^14^,^15^.

One of the difficulties faced by researchers in the area is measuring and quantifying behavioural traits. Two broad approaches to assess animal behaviour are currently used: experimental and psychometric. While the former may seem more objective, it has several limitations: (1) it often focuses on the tendency to show a specific behaviour in a specific context at the time of testing, rather than broader trait-level responses underlying behavioural expression^16^, (2) it is very resource intensive, typically taking a considerable amount of time, (3) it relies on direct access to animals, which may limit the sample sizes studied. By contrast, psychometric approaches using behavioural questionnaires (1) rapidly and relatively inexpensively measure behavioural traits across a wide range of contexts, (2) can easily be made available to carers/ owners leading to (3) high levels of participation which often leads to (4) larger sample sizes for analysis, and (5) allow sampling from a broad geographic range. However, psychometric instruments may be poorly designed and lack biological validity, so they may become simply a tool for assessing individual differences that lack convergent validity with mechanistically important determinants of behaviour. For both approaches validation and reliability tests are often unreported^17^ and this gives further concern over the reliability of a specific approach. Nonetheless, cross-species research has shown that it is possible to successfully apply adapted psychometric research methods developed for humans to the study of other species, such as the dog^18^. A questionnaire can be answered by persons who spend a significant amount of time with their dogs in a similar way to how parents report on their children. There are few validated behavioural questionnaires for dogs described in the scientific literature and broadly speaking they can be divided into two types. Some provide behavioural profiling (just like behavioural experiments), for example Serpell and Hsu’s^19^ behavioural questionnaire developed for selecting guide dogs. Other types of questionnaires focus on identifying broader underlying traits, for example Wright *et al.*’s^20^ psychometric scale to assess impulsivity in the dog.

Among the psychometric approaches developed for use in dogs, the Dog Impulsivity Assessment Scale (DIAS^20^) is currently the most thoroughly validated psychometric instrument^2^,^3^,^20^. It is composed of 18 items, which provide both an overall questionnaire score (OQS) for *impulsivity* and values for 3 underlying factors, derived from principal components analysis of the original developmental sample^20^, namely: (1) *behavioural regulation*, (2) *aggression threshold and response to novelty*, and (3) *responsiveness*. Both OQS and its facets have been found to (a) have controlled experimental behavioural test correlates in a delayed reward test (which is a behavioural experimental test to measure impulsivity)^3^, (b) correlate with low levels of serotonin and serotonin/dopamine ratio from urine samples^3^, and (c) be stable within subjects over a time frame of several years^2^. However, to date the division into 3 factors has not been replicated in any population other than the original study population. The original DIAS results published previously^20^ also suggested that there may be considerable variation in impulsivity between breeds, but the sample size per breed was too small to formally assess this.

Behavioural differences between breeds and attempts to delineate breed-typical behavioural profiles is a controversial topic, especially when it might relate to tendencies for aggressive behaviour. There is a valid concern that breed level tendencies are sometimes inappropriately generalized to make predictions about the individual^9^. Nonetheless it should be recognized that dog breeds are distinct genetic units which originated through inbreeding that express specific phenotypic traits (including specific working behaviour) and vary in behaviour^21^. Therefore, if some behavioural traits are highly heritable, behavioural differences, on average, between breeds selected for different working purposes may be expected. While some scientists emphasize that there are very specific behavioural characteristics to each breed^22^, others draw attention to the fact that the differences between individual dogs’ behaviour within a breed often exceed variation among breeds^23^. Changes in dog breeding priorities, e.g. emphasis on appearance over behavioural function in recent decades, are probably contributing to greater behavioural variability within breeds compared to between breeds^24^. Within some breeds of dog there is often now selection for different purposes, which might result in lines within a breed, for example, to perform a particular set of tasks useful for humans (working lines) or to display a set of particular morphological traits (show lines). These different functions within a breed of the modern dog might obscure breed level differences as show lines become more similar across breeds, while the working lines remain well differentiated. The significance of this refinement of the concept of the breed into specific working and show lines (i.e. the existence of biologically distinct “breeds” within a breed) appears to have been largely overlooked in the scientific literature relating to animal personality. Accordingly breed stereotyping might no longer have much predictive validity for behavioural tendencies^9^ or, at best, be a very rough starting point for predicting future performance.

The primary aim of this study was to examine the significance of this differentiation between and within breeds of dogs by examining differences in impulsivity between two breeds with large show and working lines alongside differences between lines (working versus show) across and within breeds, in order to evaluate the effects of different selective pressures on this trait. We hypothesized working-line Border Collies would be more impulsive than working-line Labrador Retrievers, given the nature of their work, but that the breeds’ behavioural tendencies would be more similar when comparing show lines, where selection for the trait may have been relaxed or converged between breeds. This would not only highlight the effects of different selective forces on impulsivity, but also help to explain why within breed behavioural variability is so great among dogs, since trait-level impulsivity can be expected to influence most behavioural tendencies. A prerequisite to this process was to establish the robustness of the method used to assess trait impulsivity. We did this by examining the potential to replicate the structure of the established psychometric instrument DIAS in a new population of dogs.

## Results

The Dog Impulsivity Assessment Scale (DIAS) plus demographic questions (Supplementary Table S1) were made available online and 1734 dog owners completed it for two breeds of dogs that are numerous in the UK in both show and working line types: Border Collies and Labrador Retrievers. After quality checks, 1495 questionnaires were suitable for analysis and only the 1161 respondents that reported their dogs to be pure bred were used for breed comparisons (see Methods). The 1161 samples included: 716 Border Collies (225 neutered males, 152 entire males, 236 neutered females, 103 entire females) and 445 Labrador Retrievers (136 neutered males, 96 entire males, 125 neutered females, 88 entire females). The dataset included pure bred dogs from work lines (n = 656, 56.45%), show lines (n = 193, 16.611%), mixed (n = 228, 19.62%) and unknown or other lines (e.g. “agility”, “pet”, n = 84, 7.23%). Age varied from 1 to 20 years. Dogs were acquired from a breeder (n = 880, 75.73%), rescue or shelter (n = 50, 4.3%), bred by the owners (n = 103, 8.86%) and other sources (n = 128, 11.02%).

### Replication of the Dog Impulsivity Assessment Scale (DIAS) structure

The structure of the DIAS^20^ was replicated with this newly acquired dataset. The 18 items of the DIAS could be grouped into 2, 3 or 4 factors according to a principal components test’s scree plot (Supplementary Figure S1). These results showed very good consistency with the results of the original study^20^, which used the 3 factor solution based on the ability to make a biological interpretation of the factors: (1) *behavioural regulation*, (2) *aggression threshold and response to novelty*, and (3) *responsiveness*. Therefore, from here on we also used the 3 factor solution. The Cronbach’s alpha reliability coefficient was also consistent across datasets (Supplementary Table S2). However, we found two differences. The item “My dog is easy to train” did not load on Factor 1 *behavioural regulation* (though it still loaded on Factor 3 *responsiveness* and therefore in the Overall Impulsivity Questionnaire Score), and the item “My dog appears to be ‘sorry’ after it has done something wrong” did not load on any Factor and was therefore eliminated from DIAS. Removal of these items improved the Cronbach’s alpha reliability coefficient, especially for *aggression threshold and response to novelty*, and *responsiveness* (Supplementary Table S2). Compared to the earlier study from Wright *et al.*^20^, the Cronbach’s alpha values either improved or were very similar. For the overall questionnaire the value was 0.74 in the original study and is 0.73 in the current study, for factor 1 (*behavioural regulation*) it was 0.82 and it is now 0.82, for factor 2 (*aggression threshold and response to novelty*) it was 0.67 and it is now 0.71 and for factor 3 (*responsiveness*) it was 0.44 and it is now 0.45 (Supplementary Table S2). This indicates good replicability of structure, even though the value for the third factor is low, which probably reflects the greater variability of scores within this factor, which is a limitation of Cronbach’s alpha as a measure of reliability^25^.

### Comparing DIAS scores between factors

We compared breed, line (work/show), sex and neuter status using Multivariate tests (MANOVA) followed by Tests of Between-Subjects effect to detect in which impulsivity scores the differences between the groups lie. Finally, we did pairwise comparisons of factors of interest that were significant in the multivariate tests by using Mann-Whitney U tests, as the data were not normally distributed.

#### Breed and line

The two breeds and their lines (including work, show, mixed and others) were different in their impulsivity levels as measured by the DIAS scores and factor scores (between breeds MANOVA: F = 5.024, p < 0.005, Supplementary Table S3, between lines MANOVA: F = 2.048, p < 0.005, Supplementary Table S3). The effect of line was significant for *impulsivity*, *behavioural regulation* and *responsiveness*, while the effect of breed was significant only for *aggression threshold and response to novelty* (Tests of Between-Subjects Effects, Table 1, Supplementary Table S4).

**Figure 1:**
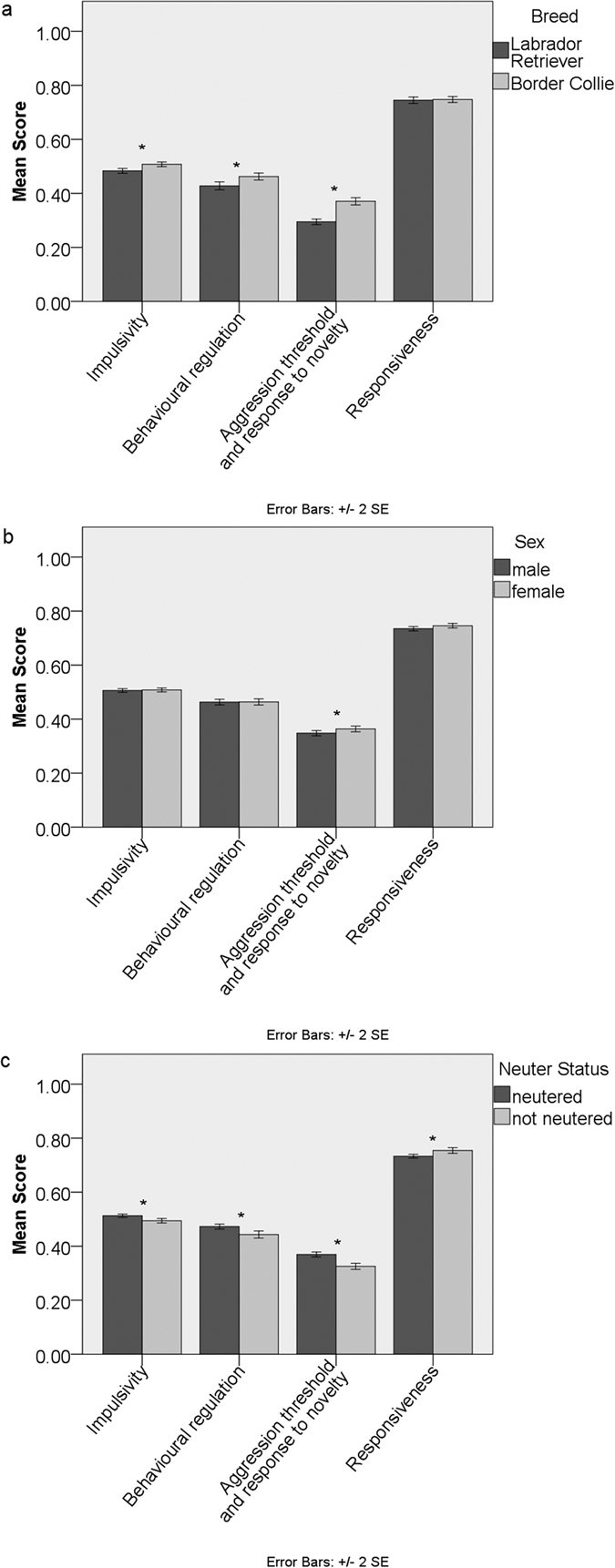
Comparison of average overall Dog Impulsivity Assessment Scale scores (DIAS) and factor scores (F1- behavioural regulation, F2- aggression threshold and response to novelty, F3- responsiveness) of Labrador Retrievers and Border Collies between: (**A**) breed, (**B**) sex and (**C**) neuter status. Significant differences (p < 0.05) Mann-Whitney U test indicated*.

When including only show and working lines and excluding mixed lines and others, Border Collies scored significantly higher than Labrador Retrievers in *impulsivity*, *behavioural regulation* and *aggression threshold and response to novelty* (Mann-Whitney U test, p < 0.005, Fig. 1, Supplementary Table S6).

**Figure 2:**
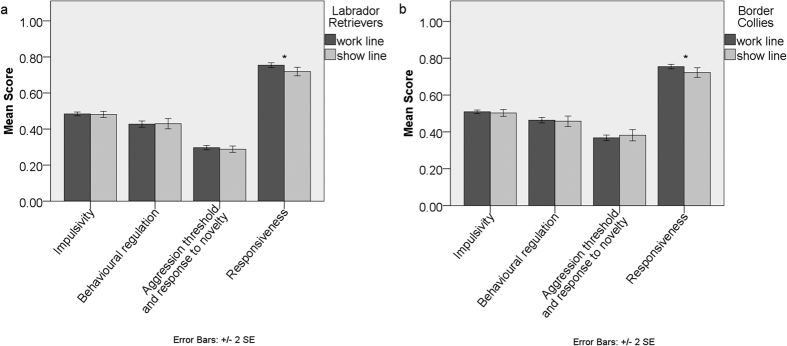
Comparison of average Dog Impulsivity Assessment Scale scores (DIAS) and factor scores (F1- behavioural regulation, F2- aggression threshold and response to novelty, F3- responsiveness) of Labrador Retrievers and Border Collies between: (**A**) work and show Labrador Retrievers, and (**B**) work and show Border Collies. Significant differences (p < 0.05) Mann-Whitney U test indicated*.

**Figure 3:**
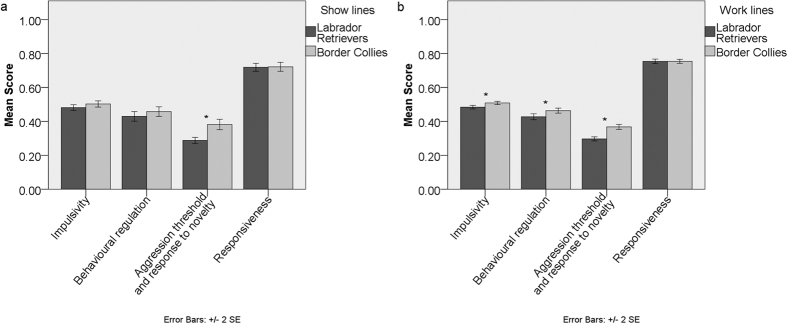
Comparison of average Dog Impulsivity Assessment Scale scores (DIAS) and factor scores (F1- behavioural regulation, F2- aggression threshold and response to novelty, F3- responsiveness) of Labrador Retrievers and Border Collies between: (**A**) show Labrador Retrievers and show Border Collies, and (**B**) working Labrador Retrievers and working Border Collies. Significant differences (p < 0.05) Mann-Whitney U test indicated*.

Work lines scored significantly higher than show lines on *responsiveness* in Labrador Retrievers, (Mann-Whitney U test, p < 0.012, Fig. 2, Supplementary Table S7) and in Border Collies as well (Mann-Whitney U test, p < 0.014, Table S6). When comparing only the work lines of the two breed breeds, working Border Collies scored significantly higher than working Labrador Retrievers in *impulsivity*, *behavioural regulation* and *aggression threshold and response to novelty* (Mann-Whitney U test, p = 0.02, p = 0.04, p < 0.001, respectively, Fig. 3, Supplementary Table S8), which are similar to the significant differences found for the breeds as a whole.

When comparing only the show lines of the two breeds, show Border Collies scored significantly higher than show Labrador Retrievers only in *aggression threshold and response to novelty* (Mann-Whitney U test, p < 0.001, respectively, Fig. 3, Supplementary Table S8). Therefore, show Labrador Retrievers and show Border Collies are more similar than work and show lines within these breeds.

#### Neuter status and sex

**Figure 4:**
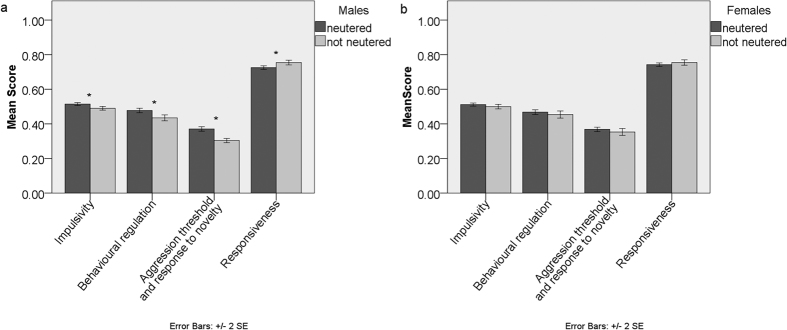
Comparison of average Dog Impulsivity Assessment Scale scores (DIAS) and factor scores (F1- behavioural regulation, F2- aggression threshold and response to novelty, F3- responsiveness) of Labrador Retrievers and Border Collies between: (**A**) Neutered and not neutered males, and (**B**) Neutered and not neutered females. Significant differences (p < 0.05) Mann-Whitney U test indicated*.

Neutered and non-neutered dogs differed on their levels of impulsivity (MANOVA: F = 2.641, p = 0.032, Supplementary Table S3) on factors *aggression threshold and response to novelty* and *responsiveness* (Tests of Between-Subjects Effects: Table 1, Supplementary Table S4). Pairwise comparison of the bigger dataset (n = 1495) showed that neutered dogs scored significantly higher than entire dogs for *impulsivity*, *behavioural regulation* and *aggression threshold and response to novelty* and significantly lower on *responsiveness* (Mann-Whitney U test, all four p values < 0.005, Fig. 1, Supplementary Table S9). However, the significant difference between entire and neutered dogs was due to neutered males scoring significantly higher in *impulsivity*, *behavioural regulation* and *aggression threshold and response to novelty* and lower in *responsiveness* in comparison to non-neutered males (p < 0.001, Fig. 4, Supplementary Table S10), while there was no significant difference between neutered and non-neutered females (Supplementary Table S10).

Even though males and females were not significantly different according to the multivariate test, there was a significant difference in the factor related to *aggression threshold and response to novelty* but with small effect size (Tests of Between-Subjects Effects: p = 0.038, Fig. 1, Supplementary Table S4), with females scoring higher than males (Mann-Whitney U test, p = 0.027, Supplementary Table S9).

#### Covariate and effect sizes

The covariate age was significant throughout (Supplementary Table S3, Supplementary Table S4). None of the interactions between factors were significant (Supplementary Table S3).

The effect size of all these tests was small, meaning that the significance of the differences found might reflect partially the large sample size (Partial Eta Squared η^2^ < 0.06, Supplementary Table S3, Supplementary Table S4), so conclusions on the biological impact of these results should be interpreted carefully.

The observed covariance matrices of the dependent variables are not equal across groups (Box’s test of Equality of Covariance Matrices F = 1678, p < 0.001), which means the risk of type 1 error is increased and significance has to be interpreted with care. The error variance of the dependent variable is not equal across groups for factor 2 *aggression threshold and response to novelty* (Levene’s Test of Equality of Error Variances F = 4716, p < 0.001, Supplementary table S5). These last two test results are very likely the product of the large sample sizes and different samples sizes across groups. However, Mann-Whitney U test shows that there are significant differences between groups and scores.

## Discussion

The use of psychometric tools, answered by proxy, for assessing behaviour in children, pets and zoo animals has been growing in the last few decades (e.g.^16^,^26^,^27^,^28^). In human psychology it has been shown that early individual differences detected through parent reports about their children are related to later personality and social development^16^. There is interest among dog breeders for detecting personality traits in puppies that will later be reflected in their adult life, which could make it easier to select dogs for specific roles, for example working and assistance dogs and dogs suitable for households with children. In addition, research on particular aspects of dog personality may help us understand corresponding aspects of human personality. Studies show that personality trait research can be applied in a cross-species manner, especially dog-human^18^. Comparative approaches among different animal species are also possible^29^. Studies on personality are relatively easy to carry out in dogs because they are widely owned, leading to large sample sizes such as the one reported here, which are not easily reached with some other species. Dogs display a wide array of behaviours and their behavioural repertoire can be assessed well by people who spend a considerable amount of time with them^18^.

Here we evaluated the effects of different selective pressures on trait impulsivity in work and show lines within two breeds. Our hypothesis that working-line Border Collies would be more impulsive than working-line Labrador Retrievers, given the nature of their work, and that the breeds would be more similar when comparing show lines, where selection for the trait may have been relaxed or converged between breeds was broadly supported. In fact, line showed significant effects on *impulsivity*, *behavioural regulation* and *responsiveness* across the data set (Table 1, Supplementary Table S2, Supplementary Table S3); this was not the case with breed, which mainly affected *aggression threshold and response to novelty*. Even though the effect size was small, meaning that the behavioural differences detected are potentially not as biologically meaningful as they may initially look, it is of clinical and social relevance (given the tendency to make breed level generalizations) that there were more differences WITHIN breeds than BETWEEN breeds (Mann-Whitney U tests, Figs 1,2 and 3, Supplementary Table S6, Supplementary Table S7, Supplementary Table S8). This indicates that line and not breed is a more general feature around which the impulsivity of pure-breed domestic dogs may be defined. This is consistent with Lofgren *et al.*^30^ who reported that in Labrador Retrievers, differentiation into work, show and pet lines was the best predictor of personality. We detected significant differences between working Labrador Retrievers and working Border Collies in *impulsivity*, *behavioural regulation* and *aggression threshold and response to novelty*. However, show Labrador Retrievers and show Border Collies displayed a significant difference in *aggression threshold and response to novelty* only (Fig. 3), which is also the factor upon which these breeds were most clearly differentiated. These results are therefore consistent with the hypothesis that the creation of a show line results in a significant loss in behavioural diversity traditionally associated with a particular breed with regards to work related behaviour. Another study^31^, has also found that show and work lines of 31 Swedish breeds are differentiating in relation to behaviour over the last century probably due to changes in selection by the breeders.

It can be speculated that the differences found are related to the different purposes of breed selection going on in the different lines which lead to unintentional secondary selection of behavioural traits, such as impulsivity. The largest difference between the working lines of both breeds was in *aggression threshold and response to novelty*. The mean *aggression threshold and response to novelty* score was higher for Border Collies, implying a lower aggression threshold and more negative responses on average for this breed compared to Labrador Retrievers. While we can speculate that higher mean *aggression threshold and response to novelty* scores in Border Collies may be the result of selection for its specific type of work (i.e. the ability to eye, stalk, chase and nip/snap at livestock, under the direction of shepherds) it is also worth noting that Collies primarily work on familiar farm land in rural areas, whereas Labradors are required to work in more variable hunting environments. Inability to cope with novelty or aggressive behaviour towards unknown people may therefore have been selected against in Labradors but not in Collies. Another study also found differences between “pastoral” dogs (where Border Collies would be included) and “gundogs” (where Labrador Retrievers would be included), in which “pastoral” dogs had higher “risk factors for aggression towards unfamiliar people outside of the household”^32^. Although they analyzed a different aspect of aggressive behaviour, both studies point towards herding dogs being more reactive in face of novelty (novel objects in the case of the DIAS^20^ or unfamiliar people in the other instance^32^).

When taking into account only Labradors, working Labrador Retrievers scored significantly higher on *responsiveness* than show Labrador Retrievers (Fig. 2). This may be expected since animals used for work purposes are selected for their ability to perform the role. However, it is also possible that these differences reflect experiential differences, as people acquiring dogs for work purposes may be more likely to work and train them accordingly. Gundogs are expected to be responsive and obedient for long periods of time while working^30^. On the other hand, owners of show dogs are probably not as demanding in the precise activity of their dogs. Lofgren *et al.*^30^ found that working Labradors scored higher than pet and show Labradors using the C-BARQ questionnaire for “trainability”, which is in accordance to our measurements of *responsiveness.*

Meanwhile, the show lines seem to be selected mostly for appearance with relaxed behaviour selection, but to some extent also for similar behaviour regardless of breed, i.e. tolerating handling by strangers, ignoring other dogs and people, standing still for longer periods of time. Hence, the convergence in impulsivity in the two breeds when considering only show lines. This not only highlights the effects of different selective forces on this trait, but also helps to explain why within breed behavioural variability is so great among dogs, since trait-level impulsivity can be expected to influence most behavioural tendencies. It would be useful to replicate this study with other breeds to determine if similar selection pressure is happening among other breeds in the show ring.

Although our findings show that there are significant differences in DIAS scores and factor scores between breeds (MANOVA: F = 5.024, p < 0.005, Fig. 1, Supplementary Table S3), which may provide some support for Svartberg’s^22^ conclusion that there are specific behavioural characteristics in each breed, our results also show that the variation within each breed is relatively high. Differences in the overall DIAS scores identified between the two breeds, although statistically significant, were numerically very small with small effect sizes. Perhaps of more practical interest is the large variation observed within each breed, since it means that individuals within both Labrador Retrievers and Border Collies might express levels of impulsivity ranging from very low to very high, making breed on its own a poor predictor of this trait. Other studies have also provided several significantly different results between different breeds and contexts, but all with substantial within-breed variation of scores (for a review see^23^). Taken together these results suggest that it is inappropriate to make predictions about an individual dog’s behavioural tendency solely based on its breed^33^. This is also consistent with Mehrkam and Wynne’s^23^ discussion of differences in individual dogs’ behaviour within a breed exceeding variations among breeds. Another practical implication of our results together with others that report large within breed variation, relates to the risk of breed-typical behaviour stereotyping, which has shaped legislative developments despite lacking scientific foundation^9^. It is the behaviour of an individual dog not the breed which is of importance when assessing the risk it poses or its desirability for re-homing, working roles or need for behavioural management.

Several online sources that advise people interested in getting a new dog list the “looking for the right breed” as an important item to take into consideration. Besides taking into account the morphological aspects of a breed, they all give advice on behaviour based on the breed, which, an increasing number of studies are showing (current work,^23^,^31^), is not a reliable measure of behaviour. This leads to unmet expectations and potentially problems with the owner-dog relationship as a result. Our results suggest that choosing the right line of dog within a breed may often be a more important factor when considering behaviour.

Secondarily, we also assessed the influence of other potentially important biological factors such as sex and neuter status, and the robustness of DIAS, by examining the potential to replicate the structure of the instrument in a new population of dogs. Little is known about the effects of neutering on dog temperament since research on neutering is sparse and not conclusive^34^. Our results showed that neutered males scored significantly higher than entire males for the factors *impulsivity*, *behavioural regulation*, *aggression threshold and response to novelty* and lower for *responsiveness* (Fig. 4). However, we do not know why animals were neutered and so cannot rule out the possibility that neutering may be the consequence rather than cause of these differences. One of the few studies that controlled for “reason for neutering”^35^ found that all but one significant difference between neutered and entire vanished, after controlling for the reason why individuals were neutered and eliminating those neutered for showing aggressive behaviour. These results indicate the importance of controlling for neuter status when considering behavioural differences between populations. More specific studies on behavioural changes associated with neutering are needed to draw more reliable conclusions of clinical relevance.

Regarding the robustness of the DIAS, the current work provided data for further validation of the instrument with a new population. In the original work^20^, 560 completed questionnaires from 107 different breeds were used for validation. The current project received more than 1700 responses relating to only two breeds. This large dataset allowed us to assess the reliability of the structure of the DIAS. We demonstrated that the structure of the DIAS is stable over different populations, while an earlier study showed that the DIAS is stable within subjects over time^2^. We are not aware of any other instrument for measuring canine/animal behavioural tendencies which has shown such a level of validity as the DIAS.

Even though we restricted this dataset to only two breeds, we obtained very similar results to when it was used with multiple breeds, indicating that the structure is generally robust, but our analysis suggested we can refine the tool by removing the item “Dog appears to be ‘sorry’ after it has done something wrong”, which in the present study did not load strongly on any of the three main factors. Personal communication with some owners that answered the questionnaire as well as other researchers that have used this tool, elicited the general comment that this question may be interpreted variably. Recent research has shown that the “guilty” or “sorry” look in dogs is most likely due to scolding from the owner rather than any awareness by the dog of a misdeed^36^,^37^,^38^ and increased understanding of this by some owners may influence response to this item. The removal of this question from the questionnaire led to an increase in the Cronbach’s alpha value from 0.44 to 0.45 (Supplementary Table S2). We recommend this amendment to the questionnaire in its future use.

## Conclusions

We found that differences between the breeds Border Collies and Labrador Retrievers are smaller than the differences between their show and work lines. Although the statistically significant differences were based on small effect sizes, the fact that the differences WITHIN breeds exceed the differences BETWEEN breed is important given practical and legal implications of breed stereotyping. Our results show that generalizations based on breed are not appropriate.

## Methods

This study was carried out in accordance to UK guidelines and was approved by the Ethics Committee of the University of Lincoln’s School of Life Sciences. Informed consent was obtained from all dog owners who participated.

### Study subjects

Data were collected from two common breeds of dog: Border Collies (n = 716) and Labrador Retrievers (n = 445). These breeds were selected as they are commonly bred and kept as pets and working animals in the UK (Kennel Club Breed Registration Statistics, 2013), and were originally selected for working purposes probably requiring different levels of impulse control (livestock herding and gundog work, respectively).

### Questionnaire: Dog Impulsivity Assessment Scale

The Dog Impulsivity Assessment Scale (DIAS) is a dog owner reported questionnaire composed of 18 behavioural items which the owner can strongly agree, mainly agree, partly agree/partly disagree, mainly disagree, or strongly disagree with. Some examples of the items are: “My dog doesn’t like to be approached or hugged”, “My dog is not keen to go into new situations”, “My dog calms down very quickly after being excited” (for the complete questionnaire and more information, see Wright *et al.*^20^). It was developed by Wright *et al.*^20^, and has been extensively validated and shown to be reliable^2^,^3^,^20^, including correlations with neurotransmitter levels and behavioural testing. It takes around 10 minutes to complete.

### Data collection

The DIAS was available for completion via an online survey site for a period of 2 weeks. The link was advertised through social media, such as Facebook groups specialized in Labrador Retrievers, Border Collies and dog training activities (e.g. AgilityNet, ObedienceUK, UKShowdogs, Gundogs), as well as breed and working dog forums.

The DIAS scores items on a 5-point Likert scale, where “strongly agree” has a score of 5 and “strongly disagree” has a score of 1 at the extremes and “partly agree/partly disagree” is in the middle. The option “don’t know / not applicable” is also available. These values are used to calculate values for an overall score and three constituent factors. Factor 1 appears to describe general *behavioural regulation*, and includes items about arousal and impulse control. Factor 2 described as *aggression threshold and response to novelty*, consists of items related to the expression of aggressive behaviour, frustration and fear-related responses to novel objects. Factor 3 is described as *responsiveness*, with items grouped together that relate to the dog’s interest in its environment and ease of training.

In addition to the DIAS, seven demographic questions were included (Supplementary Table S1). These questions were used to allow comparison between:

- Breeds (Labrador Retriever versus Border Collie)
- Work versus Show lines (the options “mixed” or “other” were also available)
- Sex (Male versus Female)
- Neuter status (Neutered versus Not-neutered)

In order to ensure dogs included were pure bred examples of their breeds, owners were asked how confident they were that their dogs were pure bred. They were also asked about their dog’s registration with the regulating bodies The Kennel Club and the International Sheepdog Society. These societies maintain and regulate the recording of pedigree so it can be assumed with a reasonable degree of confidence that dogs registered with them are pure bred. Only dogs reported to be pure bred were included in the breed and line comparison.

### Statistical analysis

Data were scored as per Wright *et al.*^20^, and data from individual respondents that had four or more missing items (i.e. “don’t know / not applicable” responses or blank) were removed from the data set (n = 1495). Those with answers “mixed”, “don’t know” or “other” for work and show lineage were not used for line and within breed comparisons (n = 849). All statistical tests were performed using IBM SPSS Statistics 22.

### Replication of the Principal Component Analysis (PCA) of the Dog Impulsivity Assessment Scale (DIAS) results based on the new dataset

In order to evaluate further the validity of DIAS, we replicated the analysis^20^ of its structure using the new dataset. Scores for the 18 DIAS items were subject to Principal Component Analysis using a varimax rotation. The Kaiser criterion (in which only factors with an eigenvalue over 1 are retained) and the Scree plot (in which the point of inflection on the graphical plot of eigenvalues is used to determine the number of factors to be retained) were considered for determining the number of factors to extract. The content matrix and item loading were checked using the criterion of suppressing items that loaded on a factor with a value less than 0.4. The replicability of the internal consistency of the 3 factors was estimated using Cronbach’s alpha as per Wright *et al.*^20^. The replicability of the Cronbach’s alpha values from the current study for each factor were accepted if they approximated those of Wright *et al.*^20^, i.e. were within +/− 0.05 of the original values.

### Comparisons of impulsivity and factor scores for demographic factors

In order to assess if there are significant differences in the DIAS scores or any of the 3 component factors scores between and within breeds, as well as between sex, neuter status and line (work, show), we used MANOVA. The dependent or outcome variables were scores on the 3 factors and the overall questionnaire score. Breed, line (show/work), sex and neuter status were the independent variables or predictors. Age was added as a covariate. Interactions between factors (breed, work/show, sex, neuter status) that best explain variation in scores of *impulsivity*, *behavioural regulation*, *aggression threshold and responses to novelty* and *responsiveness* were looked at with Tests of Between-Subjects Effects. The data was not normally distributed, however MANOVA is robust to deviations from normality^39^, therefore the post-hoc tests were done using Mann–Whitney U tests. For the variables that showed significant differences, we followed up with pairwise Mann–Whitney U tests for each demographic variable in question (i.e. Male X Female, Work X Show, etc).

## Additional Information

**How to cite this article**: Fadel, F. R. *et al.* Differences in Trait Impulsivity Indicate Diversification of Dog Breeds into Working and Show Lines. *Sci. Rep.* **6**, 22162; doi: 10.1038/srep22162 (2016).

## Supplementary Material

Supplementary Information

## Figures and Tables

**Table 1:** Variables differing significantly within one or more aspect of impulsivity (Tests of Between-Subjects Effects, p < 0.05, Supplementary Table S4) as measured by the Dog Impulsivity Assessment Scale (DIAS).

OQS Overall QuestionnaireScore - *mpulsivity*	Factor 1*Behavioural Regulation*	Factor 2*Aggression Threshold and Response to Novelty*	Factor 3*Responsiveness*
Age (covariate)	Age (covariate)		Age (covariate)
		Breed	
Line (Work/Show)	Line (Work/Show)		Line (Work/Show)
		Sex	
		Neuter Status	Neuter Status
