# Supplementary material for: Differences in Trait Impulsivity Indicate Diversification of Dog Breeds into Working and Show Lines
Source: Sci Rep. 2016 Mar 10;6:22162. doi: 10.1038/srep22162 (PMC4785826; doi:10.1038/srep22162)
Supplement: Supplementary Information [file srep22162-s1.doc]

**DIFFERENCES IN TRAIT IMPULSIVITY INDICATE DIVERSIFICATION OF DOG BREEDS INTO WORKING AND SHOW LINES**

Fernanda Ruiz FADEL^a^ *, Patricia DRISCOLL^a^, Malgorzata PILOT^a^, Hannah WRIGHT^a^, Helen ZULCH^a^, Daniel MILLS^a^

^a^ University of Lincoln, School of Life Sciences, Joseph Banks Laboratories, Lincoln, LN6 7DL, UK

* ffadel@lincoln.ac.uk

# Supplementary Information


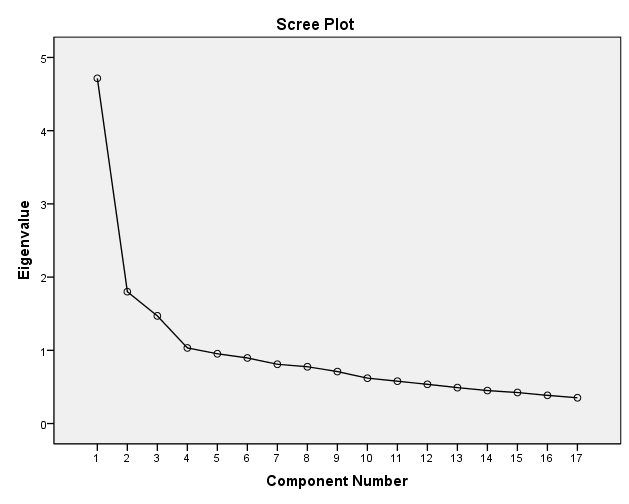


Supplementary Figure S1 Scree Plot of the components identified in the Principal Components Analysis of the 17 items from the Dog Impulsivity Assessment Scale (DIAS) using the new dataset.

Supplementary Table S1 Demographic items included in the questionnaire together with the Dog Impulsivity Assessment Scale (DIAS) to be answered by dog owners.

| What breed is your dog?   - Labrador Retriever - Border Collie / Working Sheepdog   Do you consider your dog to be:   - Predominantly working bred i.e. bred from lines that work livestock (Border Collies) or gundog work (Labradors) - Predominantly show bred (i.e. bred from dogs intended to be shown in the breed ring) - Mixed show and working lines - Don't know - Other (please specify)   Where did you get this dog from?   - From a breeder - From a rescue or shelter - Bred by yourself - Other (please specify)   What sex is your dog?   - Male - Female   What is this dog's neuter status?   - Neutered - Not neutered   How old is this dog? If you don't know, please make as close an estimate as possible  _________  How would you describe your dog's level of training?   - Little or none - Moderate amount - Lots of training |
| --- |

Supplementary Table S2 Cronbach’s alpha reliability coefficient for repeated 3-factor solution (from Principal Components Analysis) of the Dog Impulsivity Assessment Scale (DIAS) from Wright et al. 2011 and the current work.

|  | *Impulsivity* –  Overall Questionnaire Score (OQS) | *Behavioural Regulation* – Factor 1 (F1) | *Aggression Threshold and response to Novelty* – Factor 2 (F2) | *Responsiveness* – Factor 3 (F3) |
| --- | --- | --- | --- | --- |
| Wright at al., 2011 | 0.74 | 0.82 | 0.67 | 0.44 |
| Current Dataset | 0.73 | 0.82 | 0.71 | 0.45 |

Supplementary Table S3 MANOVA to test for interactions between the factors, with four fixed factors (breed, sex, work/show, neuter status) for Dog Impulsivity Assessment Scale (DIAS) scores and each of the 3 Factors scores with age as a covariate. Significant values from Multivariate Test Wilk’s Lambda in bold.

|  | Value | F | Hypothesis df | Error df | Sig. | Partial Eta Squared |
| --- | --- | --- | --- | --- | --- | --- |
| Age (covariate) | .976 | 6.934^b^ | 4.000 | 1117.000 | **<.001** | .024 |
| Breed | .982 | 5.024^b^ | 4.000 | 1117.000 | **.001** | .018 |
| Sex | .993 | 1.990^b^ | 4.000 | 1117.000 | .094 | .007 |
| Work/Show | .971 | 2.048 | 16.000 | 3413.129 | **.008** | .007 |
| Neuter Status | .991 | 2.641^b^ | 4.000 | 1117.000 | **.032** | .009 |
| Breed * Sex | .996 | 1.204^b^ | 4.000 | 1117.000 | .307 | .004 |
| Breed * Work/Show | .991 | .667 | 16.000 | 3413.129 | .829 | .002 |
| Breed * Neuter Status | .994 | 1.823^b^ | 4.000 | 1117.000 | .122 | .006 |
| Sex * Work/Show | .985 | 1.083 | 16.000 | 3413.129 | .365 | .004 |
| Sex * Neuter Status | .995 | 1.299^b^ | 4.000 | 1117.000 | .269 | .005 |
| Work/Show * Neuter Status | .983 | 1.181 | 16.000 | 3413.129 | .275 | .004 |
| Breed * Sex * Work/Show | .984 | 1.144 | 16.000 | 3413.129 | .307 | .004 |
| Breed * Sex * Neuter Status | .998 | .461^b^ | 4.000 | 1117.000 | .765 | .002 |
| Breed * Work/Show * Neuter Status | .984 | 1.161 | 16.000 | 3413.129 | .292 | .004 |
| Sex * Work/Show * Neuter Status | .995 | .356 | 16.000 | 3413.129 | .991 | .001 |
| Breed * Sex * Work/Show *Neuter Status | .992 | .575 | 16.000 | 3413.129 | .905 | .002 |

Supplementary Table S4 Tests of Between-Subjects Effects to check in which scores the factors are different: Dog Impulsivity Assessment Scale (DIAS) scores and each of the 3 Factors scores for each demographic factor (Breed, Sex, Line, Neuter-Status), age as a covariate. Significant values in bold.

| Source | Dependent Variable | Type III Sum of Squares | df | Mean Square | F | Sig. | Partial Eta Squared |
| --- | --- | --- | --- | --- | --- | --- | --- |
| Age (covariate) | OQS *impulsivity* | .104 | 1 | .104 | 12.750 | **<.001** | .011 |
|  | F1 *behavioural regulation* | .235 | 1 | .235 | 11.629 | **.001** | .010 |
|  | F2 *aggression threshold and response to novelty* | .001 | 1 | .001 | .044 | .834 | .000 |
|  | F3 *responsiveness* | .170 | 1 | .170 | 13.150 | **<.001** | .012 |
| Breed | OQS *impulsivity* | .022 | 1 | .022 | 2.698 | .101 | .002 |
|  | F1 *behavioural regulation* | .018 | 1 | .018 | .901 | .343 | .001 |
|  | F2 *aggression threshold and response to novelty* | .259 | 1 | .259 | 15.779 | **<.001** | .014 |
|  | F3 *responsiveness* | .001 | 1 | .001 | .097 | .755 | .000 |
| Work/Show | OQS *impulsivity* | .167 | 4 | .042 | 5.097 | **<.001** | .018 |
|  | F1 *behavioural regulation* | .357 | 4 | .089 | 4.419 | **.002** | .016 |
|  | F2 *aggression threshold and response to novelty* | .067 | 4 | .017 | 1.027 | .392 | .004 |
|  | F3 *responsiveness* | .125 | 4 | .031 | 2.419 | **.047** | .009 |
| Sex | OQS *impulsivity* | .003 | 1 | .003 | .409 | .522 | .000 |
|  | F1 *behavioural regulation* | .015 | 1 | .015 | .757 | .384 | .001 |
|  | F2 *aggression threshold and response to novelty* | .071 | 1 | .071 | 4.328 | **.038** | .004 |
|  | F3 *responsiveness* | .000 | 1 | .000 | .033 | .856 | .000 |
| Neuter Status | OQS *impulsivity* | .004 | 1 | .004 | .459 | .498 | .000 |
|  | F1 *behavioural regulation* | .003 | 1 | .003 | .130 | .719 | .000 |
|  | F2 *aggression threshold and response to novelty* | .090 | 1 | .090 | 5.478 | **.019** | .005 |
|  | F3 *responsiveness* | .058 | 1 | .058 | 4.507 | **.034** | .004 |

Supplementary Table S5 Levene’s Test of Equality of Error Variances for the multivariate tests from Supplementary Table S3 and Supplementary Table S4. Tests the null hypothesis that the error variance of the dependent variables (scores for OQS, F1, F2 and F3) is equal across groups (breed, line, sex, neuter status). Significant values in bold.

|  | F | Sig. |
| --- | --- | --- |
| OQS *impulsivity* | 1.115 | 0.290 |
| F1 *behavioural regulation* | 1.026 | 0.427 |
| F2 *aggression threshold and response to novelty* | 4.716 | **<0.001** |
| F3 *responsiveness* | 1.142 | 0.255 |

Supplementary Table S6 Mean and median Dog Impulsivity Assessment Scale (DIAS) scores of Labrador Retrievers and Border Collies and P-value from Mann-Whitney U test between breed. Includes dogs from work and show lineage only. Significant values in bold.

|  | OQS (SD) *impulsivity* | F1 (SD) *behavioural regulation* | F2 (SD) *aggression threshold and response to novelty* | F3 (SD) *responsiveness* |
| --- | --- | --- | --- | --- |
| Overall n=849 | Mean=0.5 (0.09)  Median=0.49 | Mean=0.45 (0.14)  Median=0.44 | Mean=0.34 (0.14)  Median=0.28 | Mean=0.75 (0.12)  Median=0.76 |
| Pure bred Labrador Retrievers n=334 | Mean=0.48 (0.08)  Median=0.47 | Mean=0.43 (0.14)  Median=0.42 | Mean=0.30 (0.09)  Median=0.28 | Mean=0.75 (0.11)  Median=0.76 |
| Pure bred Border Collies n=515 | Mean=0.51 (0.1)  Median=0.50 | Mean=0.46 (0.15)  Median=0.44 | Mean=0.37 (0.15)  Median=0.32 | Mean=0.75 (0.12)  Median=0.76 |
| Breed comparison | **U=73959.5**  **p=0.001** | **U=74884.5**  **p=0.001** | **U=61625**  **p<0.001** | U=84256  p=0.614 |

Supplementary Table S7 Mean and Median Dog Impulsivity Assessment Scale (DIAS) scores of work and show lines of Labrador Retrievers and Border collies and *P*-value from Mann-Whitney U test between: lines in Labrador Retrievers, and lines in Border Collies. Significant values in bold.

| N=849 pure bred and of known lineage | OQS (SD)  *impulsivity* | F1 (SD) *behavioural regulation* | F2 (SD) *aggression threshold and response to novelty* | F3 (SD) *responsiveness* |
| --- | --- | --- | --- | --- |
| Overall Labrador Retrievers n=334 | Mean=0.48 (0.08)  Median=0.54 | Mean=0.43 (0.14)  Median=0.52 | Mean=0.30 (0.09)  Median=0.32 | Mean=0.75 (0.11)  Median=0.8 |
| Work Labrador Retrievers n=246 | Mean=0.48(0.08)  Median=0.48 | Mean=0.43(0.14)  Median=0.42 | Mean=0.30(0.1)  Meadian=0.28 | Mean=0.75(0.11)  Meadian=0.76 |
| Show Labrador Retrievers n=88 | Mean=0.48(0.08)  Median=0.47 | Mean=0.43(0.13)  Median=0.42 | Mean=0.29(0.08)  Median=0.28 | Mean=0.72(0.11)  Median=0.72 |
| Line comparison in Labrador Retrievers | U=10689.5  p=0.863 | U=10729.5  p=0.903 | U=10464.5  p=0.639 | **U=8889.5**  **p=0.012** |
| Overall Border Collies n=515 | Mean=0.51 (0.1)  Median=0.50 | Mean=0.46 (0.15)  Median=0.44 | Mean=0.37 (0.15)  Median=0.32 | Mean=0.75 (0.12)  Median=0.76 |
| Work Border Collies n=410 | Mean=0.51(0.1)  Median=0.51 | Mean=0.46(0.15)  Median=0.44 | Mean=0.37(0.15)  Median=0.32 | Mean=0.75(0.12)  Median=0.76 |
| Show Border Collies n=105 | Mean=0.50(0.1)  Median=0.49 | Mean=0.46(0.15)  Median=0.44 | Mean=0.38(0.16)  Median=0.36 | Mean=0.72(0.14)  Median=0.72 |
| Line comparison in Border Collies | U=20434  p=0.422 | U=21078.5  p=0.743 | U=20415.5  p=0.412 | **U=18259**  **p=0.016** |

Supplementary Table S8 Mean and Median Dog Impulsivity Assessment Scale (DIAS) scores of work and show lines of dogs from the breeds Labrador Retriever and Border Collie and *P*-value from Mann-Whitney U test between: working Labrador Retrievers and working Border collies; and show Labrador Retrievers and show Border collies. Significant values in bold.

| N=849 pure bred and of known lineage | OQS (SD) *impulsivity* | F1 (SD) *behavioural regulation* | F2 (SD) *aggression threshold and response to novelty* | F3 (SD) *responsiveness* |
| --- | --- | --- | --- | --- |
| Overall work lines n=656 | Mean=0.50(0.01)  Median=0.49 | Mean=0.45(0.16)  Median=0.44 | Mean=0.34(0.14)  Median=0.28 | Mean=0.75(0.12)  Median=0.76 |
| Work Labrador Retrievers n=246 | Mean=0.48(0.08)  Median=0.48 | Mean=0.43(0.14)  Median=0.42 | Mean=0.30(0.1)  Meadian=0.28 | Mean=0.75(0.11)  Meadian=0.76 |
| Work Border Collies n=410 | Mean=0.51(0.1)  Median=0.51 | Mean=0.46(0.15)  Median=0.44 | Mean=0.37(0.15)  Median=0.32 | Mean=0.75(0.12)  Median=0.76 |
| Work line comparison between Labradors and Collies | **U=43141.5**  **p=0.02** | **U=43658.5**  **p=0.04** | **U=37170**  **p<0.001** | U=49934  p=0.832 |
| Overall show lines n=193 | Mean=0.49(0.09)  Median=0.48 | Mean=0.44(0.14)  Median=0.42 | Mean=0.34(0.14)  Median=0.28 | Mean=0.72(0.13)  Median=0.72 |
| Show Labrador Retrievers n=88 | Mean=0.48(0.08)  Median=0.47 | Mean=0.43(0.13)  Median=0.42 | Mean=0.29(0.08)  Median=0.28 | Mean=0.72(0.11)  Median=0.72 |
| Show Border Collies n=105 | Mean=0.50(0.1)  Median=0.49 | Mean=0.46(0.15)  Median=0.44 | Mean=0.38(0.16)  Median=0.36 | Mean=0.72(0.14)  Median=0.72 |
| Line comparison between Labradors and Collies | U=4085.5  p=0.166 | U=4110  p=0.187 | **U=2980**  **p<0.001** | U=4586  p=0.930 |

Supplementary Table S9 Mean and Median Dog Impulsivity Assessment Scale (DIAS) scores of male and female dogs, and neutered and not neutered dogs, and P-value from Mann-Whitney U test between sex and neuter status. Includes dogs from breeds Border Collies, Labrador Retriever and crosses including one of these breeds. Significant values in bold.

|  | OQS (SD) *impulsivity* | F1 (SD) *behavioural regulation* | F2 (SD) *aggression threshold and response to novelty* | F3 (SD) *responsiveness* |
| --- | --- | --- | --- | --- |
| Overall n=1495 | Mean=0.51(0.10)  Median=0.50 | Mean=0.46 (0.15)  Median=0.44 | Mean=0.35 (0.14)  Median=0.32 | Mean=0.74 (0.11)  Median=076 |
| Male | Mean=0.51 (0.09)  Median=0.50 | Mean=0.46 (0.14)  Median=0.44 | Mean=0.35 (0.14)  Median=0.32 | Mean=0.74 (0.12)  Median=0.72 |
| Female | Mean=0.51 (0.10)  Median=0.50 | Mean=0.46 (0.15)  Median=0.44 | Mean=0.36 (0.14)  Median=0.32 | Mean=0.75 (0.11)  Median=0.76 |
| Sex comparison | U=276059  p=0.772 | U=277988.5  p=0.953 | **U=260173**  **p=0.027** | U=264556  p=0.093 |
| Neutered | Mean=0.51 (0.10)  Median=0.51 | Mean=0.48 (0.16)  Median=0.46 | Mean=0.37 (0.14)  Median=0.32 | Mean=0.76 (0.12)  Median=0.72 |
| Not neutered | Mean=0.49 (0.09)  Median=0.49 | Mean=0.45 (0.15)  Median=0.42 | Mean=0.33 (0.12)  Median=0.28 | Mean=0.78 (0.12)  Median=0.76 |
| Neuter status comparison | **U=217895**  **p=0.002** | **U=215822**  **p=0.001** | **U=197838**  **p<0.001** | **U=217385**  **p<0.001** |

Supplementary Table S10 Mean Dog Impulsivity Assessment Scale (DIAS) scores of male and female dogs and *P*-value from Mann-Whitney U test between: neutered and not neutered males; and neutered and not neutered females, 1495 dogs included. Includes dogs from breeds Border Collies, Labrador Retriever and crosses including one of these breeds. Significant values in bold.

| N=1495 | OQS (SD) *impulsivity* | F1 (SD) *behavioural regulation* | F2 (SD) *aggression threshold and response to novelty* | F3 (SD) *responsiveness* |
| --- | --- | --- | --- | --- |
| Overall Males | Mean=0.51 (0.09)  Median=0.50 | Mean=0.46 (0.14)  Median=0.44 | Mean=0.35 (0.14)  Median=0.32 | Mean=0.74 (0.12)  Median=0.72 |
| Neutered males | Mean=0.51 (0.10)  Median=0.51 | Mean=0.48 (0.15)  Median0.47 | Mean=0.37 (0.15)  Median=0.33 | Mean=0.73 (0.12)  Median=0.72 |
| Not neutered males | Mean=0.49 (0.08)  Median=0.48 | Mean=0.43 (0.14)  Median=0.42 | Mean=0.30 (0.10)  Median=0.28 | Mean=0.75 (0.11)  Median=0.76 |
| Neuter status comparison in males | **U=59892**  **p=0.001** | **U=58531**  **p<0.001** | **U=50624.5**  **p<0.001** | **U=59731**  **p=0.001** |
| Overall Females | Mean=0.51 (0.10)  Median=0.50 | Mean=0.46 (0.15)  Median=0.44 | Mean=0.36 (0.14)  Median=0.32 | Mean=0.75 (0.11)  Median=0.76 |
| Neutered females | Mean=0.51 (0.1)  Median=0.51 | Mean=0.47 (0.15)  Median=0.44 | Mean=0.37 (0.14)  Median=0.32 | Mean=0.74 (0.11)  Median=.76 |
| Not neutered females | Mean=0.49 (0.10)  Median=0.49 | Mean=0.45 (0.15)  Median=0.44 | Mean=0.35 (0.14)  Median=0.32 | Mean=0.76 (0.11)  Median=0.76 |
| Neuter status comparison in females | U=49056  p=0.238 | U=49307  p=0.280 | U=48167.5  p=0.122 | U=48777  p=0.193 |
